# The European Brain Council: toward sustained and better coordinated brain research in Europe

**DOI:** 10.3325/cmj.2019.60.150

**Published:** 2019-04

**Authors:** Monica Di Luca, Frederic Destrebecq, Giovanni Esposito

**Affiliations:** European Brain Council *frde@braincouncil.eu*

The European Brain Council (EBC) serves as a pan-European platform, bringing together brain researchers, patient advocates, scientists, and clinicians around a common vision for the “brain space.” At the heart of our advocacy work are accelerating brain research, raising awareness of the challenges associated with brain disorders, reducing stigma and discrimination, and supporting science. Our mission revolves around promoting brain research and brain health at the European level to the benefit of people living with neurological and psychiatric disorders.

Brain disorders are highly prevalent diseases that impose a tremendous burden on European society. In 2010, more than 1 in 3 European citizens were affected by a mental or neurological disease (1). Brain disorders are responsible for the highest number of years lost due to disability at the WHO Europe level. The social but also economic burden of these conditions is staggering. Health economists have estimated that brain disorders alone amount to about 45% of the annual health budget in Europe, totaling to around €800 billion every year (2).

Above all, the burden of diseases is mainly linked to inadequate care or a lack of access to care. Out of 10 people living with a brain condition, from 3 to 8 do not receive adequate treatment even when it is available (3). In order to address this “treatment gap,” the EBC conducted a study on the value of treatment for brain disorders in Europe (3). Covering a range of mental and nervous system disorders, the study examined health gains and socio-economic impacts arising from early diagnosis and timely intervention strategies. The benefits obtained from best health interventions were compared with those obtained from the current care or lack thereof. These health gains ranged from increased survival rates, reduced complications and disability, improved quality of life, and reduced treatment costs. Among other policies, the report recommended sustained funding into basic, clinical, and translational brain research.

In order to meet the challenges posed by brain disorders, EBC developed a consensus document setting out priorities for brain research in Europe, whose third edition was released in 2016 (4). This document encapsulates the vision from the patient community as well as the scientific and clinical societies under the EBC aegis to expand brain research in Europe. While it recognizes the efforts and achievements made in the domain, it also restates clear expectations and proposes future research programs. As a whole, it addressed the main challenges in the field in an integrated manner in order to ensure increased support to basic and clinical research as well as translation of research breakthroughs into practice. It also called for optimization of funding opportunities and research efforts with the final aim of overcoming fragmentation and duplication.

After the European Commission released the proposal for the new EU framework program for research and innovation entitled “Horizon Europe,” EBC has been encouraging EU institutions to live up to the expectation of doubling the total budget of the program, as proposed in the report of the high-level group on maximizing the impact of EU research and innovation programs (5). In that same report, the high-level group also recommended “a mission-oriented impact-focused approach to address global challenges” (5). Encouraged by the inclusion of this idea in the high-level group report (“understanding and enhancing the brain by 2030”) (5), EBC proposed policy makers to endorse its brain mission (to be published). To date, it is unclear how the proposed missions are going to take shape since political discussions are still under way on Horizon Europe itself, and the implementation of its various elements. Within this context, EBC has continuously campaigned for:

- A robust funding of the total program. The European Commission proposed to allocate €94.1 billion to the Horizon Europe program. EBC subsequently called on the European Commission, European Parliament, and the Council of the EU to increase the budget to €120 billion.

- Increased funding and sound structuring of the health cluster. The EU program is subdivided into pillars, clusters, areas of intervention, and broad lines. It has been EBC’s priority to get not only increased funding for the “health cluster” under the second pillar of the program (“Global Challenges and Industrial Competitiveness”), but also to ensure the creation of an appropriate and functioning structure that would enable brain research to be prioritized and efficiently addressed.

- The adoption of a brain mission. This idea, suggested by the high-level group, was taken on board of the program proposal and is currently looked into by the European Commission, European Parliament, and the EU member states. The Brain Mission, as proposed by EBC, is aimed to serve as a guiding paper in this process (6).

Amidst these political discussions, in November 2018 the EBC launched an EU-funded project The European Brain Research Area (EBRA, *www.ebra.eu*) (Grant agreement No 825348). The overall idea underpinning EBRA is to coordinate and synergize brain research projects and initiatives at both European and global level in order to foster the efficiency of efforts in brain research. EBRA brings together the brain research community at large, the major brain research initiatives, research funding networks, and all relevant stakeholders at the European level and beyond, to ensure the overall delivery of the research potential. Therefore, EBRA was created as platform that would catalyze coordination between brain researchers, clinicians, patients, governments, funders, and public institutions, while fostering global initiatives. EBRA consortium is led by EBC and brings together the Joint Programming on Neurodegenerative Diseases, the Human Brain Project, and the ERANET Neuron. For the next 3 years, the Consortium will work to align and coordinate research strategies across European and global brain initiatives; facilitate the launching of research projects in specific areas in active clusters, and help them to collaborate effectively, share data, and access research infrastructures; and increase the brain research visibility and promote the uptake of EBRA results to key stakeholders.

Brain research in Europe is a rapidly evolving field, and it has always been at the forefront of science. Nonetheless, the inherent complexity of the nervous system has made our translational capacity more challenging, hence requiring a higher level of integration. EBC, through its EBRA project, remains committed to supporting the strategic vision of favoring a sustained open research community, with high level of collaboration and knowledge-sharing across the borders of various disciplines, to improve the quality of life of all citizens living with a brain condition in Europe.

## References

[1] Wittchen HU, Jacobi F, Rehm J, Gustavsson A, Svensson M, Jönsson B (2011). The size and burden of mental disorders and other disorders of the brain in Europe 2010.. Eur Neuropsychopharmacol.

[2] Gustavsson A, Svensson M, Jacobi F, Allgulander C, Alonso J, Beghi E (2011). Cost of disorders of the brain in Europe 2010.. Eur Neuropsychopharmacol.

[3] European Brain Council. EBC Policy White Paper: The Value of Treatment for Brain Disorders. Available from: https://www.braincouncil.eu/wp-content/uploads/2017/06/EBC_white_policy_paper_DEF26072017_Low.pdf. Accessed: April 4, 2019.

[4] Morris R, Oertel W, Gaebel W, Goodwin G, Little A, Montellano P (2016). Consensus Statement on European Brain Research: the need to expand brain research in Europe – 2015.. Eur J Neurosci.

[5] Lamy P, Brudermüller M, Ferguson M, Friis L, Garmendia C, Gray I, et al. LAB – FAB – APP Investing in the European future we want. 2017. Available from: https://www.ecsite.eu/sites/default/files/lab-fab-app.pdf. Accessed: April 4, 2019.

[6] European Brain Council. Brain mission. Available from*:* https://www.braincouncil.eu/wp-content/uploads/2018/04/Brain-Mission-Final-v2.pdf. Accessed: April 11, 2019.

